# Assessing recall of personal sun exposure by integrating UV dosimeter and self-reported data with a network flow framework

**DOI:** 10.1371/journal.pone.0225371

**Published:** 2019-12-04

**Authors:** Nabil Alshurafa, Jayalakshmi Jain, Tammy K. Stump, Bonnie Spring, June K. Robinson

**Affiliations:** 1 Department of Preventive Medicine, Northwestern University, Chicago, Illinois, United States of America; 2 Department of Computer Science, Northwestern University, Evanston, Illinois, United States of America; 3 Department of Electrical and Computer Engineering, Northwestern University, Evanston, Illinois, United States of America; 4 Robert H. Lurie Comprehensive Cancer Center, Northwestern University, Chicago, Illinois, United States of America; 5 Department of Dermatology, Northwestern University Feinberg School of Medicine, Chicago, Illinois, United States of America; University of Murcia, SPAIN

## Abstract

**Background:**

Melanoma survivors often do not engage in adequate sun protection, leading to sunburn and increasing their risk of future melanomas. Melanoma survivors do not accurately recall the extent of sun exposure they have received, thus, they may be unaware of their personal UV exposure, and this lack of awareness may contribute towards failure to change behavior. As a means of determining behavioral accuracy of recall of sun exposure, this study compared subjective self-reports of time outdoors to an objective wearable sensor. Analysis of the meaningful discrepancies between the self-report and sensor measures of time outdoors was made possible by using a network flow algorithm to align sun exposure events recorded by both measures. Aligning the two measures provides the opportunity to more accurately evaluate false positive and false negative self-reports of behavior and understand participant tendencies to over- and under-report behavior.

**Methods:**

39 melanoma survivors wore an ultraviolet light (UV) sensor on their chest while outdoors for 10 consecutive summer days and provided an end-of-day subjective self-report of their behavior while outdoors. A Network Flow Alignment framework was used to align self-report and objective UV sensor data to correct misalignment. The frequency and time of day of under- and over-reporting were identified.

**Findings:**

For the 269 days assessed, the proposed framework showed a significant increase in the Jaccard coefficient (i.e. a measure of similarity between self-report and UV sensor data) by 63.64% (*p* < .001), and significant reduction in false negative minutes by 34.43% (*p* < .001). Following alignment of the measures, under-reporting of sun exposure time occurred on 51% of the days analyzed and more participants tended to under-report than to over-report sun exposure time. Rates of under-reporting of sun exposure were highest for events that began from 12-1pm, and second-highest from 5-6pm.

**Conclusion:**

These discrepancies may reflect lack of accurate recall of sun exposure during times of peak sun intensity (10am–2pm) that could ultimately increase the risk of developing melanoma. This research provides technical contributions to the field of wearable computing, activity recognition, and identifies actionable times to improve participants’ perception of their sun exposure.

## Introduction

Melanoma survivors are at risk to develop another melanoma [1], and the same patterns of sun exposure that may have caused the initial melanoma contribute to the risk for a second melanoma [2]. Despite awareness of the risk of developing another melanoma and the benefit of sun protection in reducing that risk [3], melanoma survivors often do not engage in adequate sun protection, leading to sunburn [4]. Potentially contributing to inadequate use of sun protection could be a low understanding of ultraviolet (UV) radiation exposure [5]. Furthermore, melanoma survivors who initiate reduced sun exposure and increased use of sunscreens in the first summer after diagnosis, do not maintain these changes three years later [6]. This lack of recognition of personal UV exposure is critical to examine because knowledge of and attitudes about current levels of behavior contribute prominently to effective behavior change [7, 8]. In many health behavior change theories [9, 10] and in self-discrepancy theory [11], knowledge of one’s own behavior is important for assessing goal progress [12, 13] and for developing a sense of mastery and self-efficacy [14], which underlies long-term behavior change. Thus, if a person does not have an accurate awareness of their own behavior (here, assessed by accuracy of recalling time spent outdoors), they may be less motivated and engaged with protecting their skin from the sun, which can lead to disengagement with sun protection during outdoor activities.

**Fig 1:**
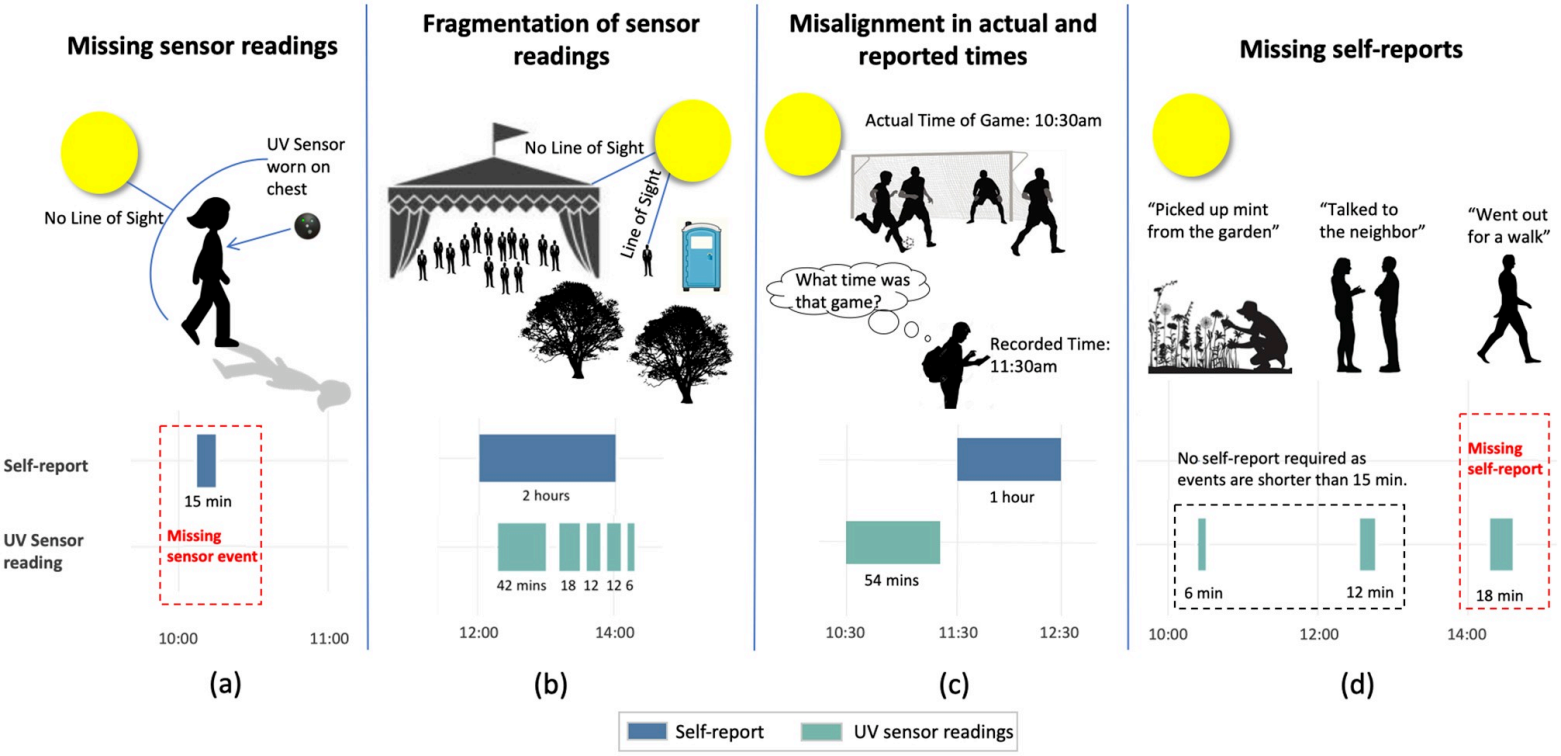
Challenges in data collected from the sensor and self-report. (a) The person is in the sun but the UV sensor is shaded by their body. No data is collected by the sensor. (b) The person reports going to an outdoor wedding but goes in and out of the tent to use the bathroom. The sensor reading is fragmented in this case. (c) The person incorrectly recollects the time of the soccer game. The actual and reported times do not align. (d) The person goes out for a walk and forgets to report their sun exposure time even though it was greater than 15 minutes in duration.

Recall of personal UV exposure can be assessed by comparing self-reports of time outdoors to outdoor time assessed by objective, wearable sensors. While comparing these two measures, it is important to evaluate and mitigate error with the sensor measure. Objective wearable sensors are also prone to different types of errors including reporting inaccuracies in uncontrolled environments (such as having the sensor facing away from the sun), lack of adherence to wearing the device, and low battery lifetime. (Fig 1) Thus, the sensor and self-report measures of time outdoors can be discrepant, in part, due to idiosyncrasies in the data collection methods rather than due to a meaningful lack of recall on the part of users. Thus, to properly compare the two measures, an algorithm is needed that aligns self-report and sensor measure (i.e. temporal synchronization or matching of self-report with its nearest viable sensor measure), considering the possible sources of error in both measures. This alignment algorithm can provide accurate understanding of when (e.g. early morning) disagreement occurs, which can guide the appropriate design of tools to effectively assist self-reflection about sun exposure and improve protection by patients at-risk to develop melanoma.

Our optimal network-flow alignment framework combined self-report and sensor data to obtain an accurate estimate of personal UV sun exposure. Our hypothesis is that the alignment will reveal discrepancies between self-report and sensor data, which could indicate when a melanoma survivor lacks accurate recall and awareness of their own behavior. In summary, in order to inform future sun exposure interventions, this project aimed to: 1) align self-report and sensor-assessed outdoor time with the intention of identifying event-level sun protection during outdoor activities, 2) evaluate meaningful discrepancies between sensors and self-reports (which may indicate recall errors and lack of behavioral awareness), and 3) assess willingness and adherence to wearing UV sensors.

## Materials and methods

### Study overview

**Fig 2:**
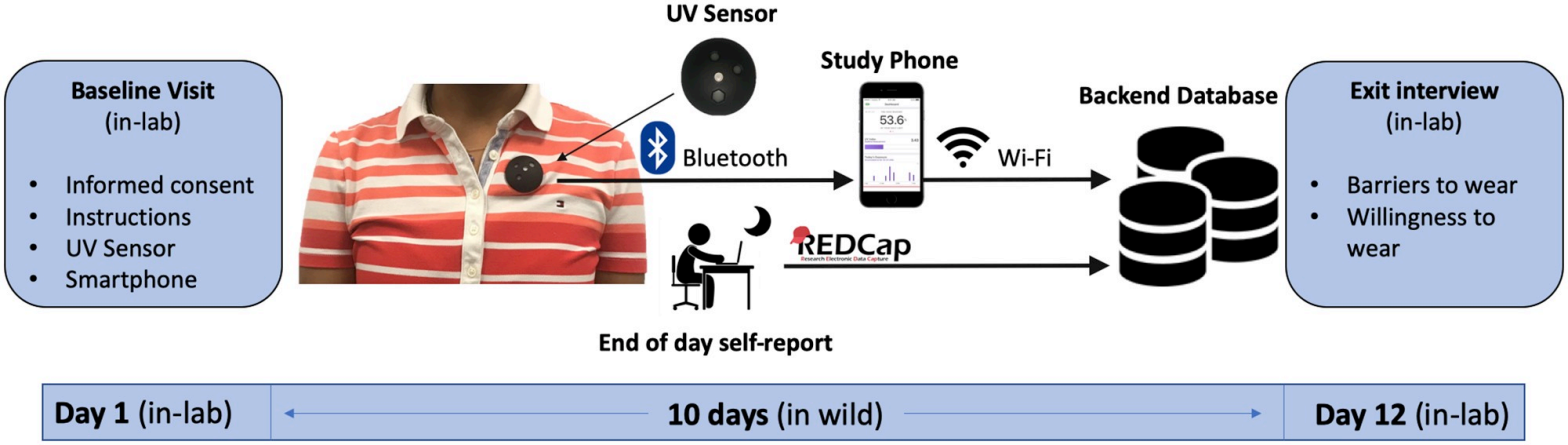
Schematic overview of the study.

Adult melanoma survivors were enrolled in a 10-day study. Participants without daily access to a computer and wireless internet were excluded from the study. They were requested to visit the laboratory twice. At the start of the baseline visit, participants provided written informed consent, and received a smartphone and a UV sensor (Shade ^®^ v1, YouV Labs Inc., NY) [15]. The study smartphone was only used to upload the data, and participants did not need to carry the device with them all day. They were emailed a link containing the Daily Minutes of Unprotected Sun Exposure (MUSE) Inventory self-report questionnaire to report their outdoor activities and sun protection habits at the end of each day [16]. Participants were sent daily reminders and instructions for performing the data upload and completing the questionnaire. The survey provided space to record problems with wearing the device or performing data uploads. Participants received a $100 gift card as compensation at the end of the study when the devices were returned. There was also an exit interview conducted at the end of the study. The exit interviews with the 39 participants were audio recorded. Two authors identified themes detected across most of the participants and logged the number of times a theme was mentioned by a participant. All visits were completed in the Midwest between July and September 2017. A schematic overview of the study is shown in Fig 2.

### Ethical considerations

This study was approved by the Northwestern University Institutional Review Board (STU00201983) and the protocol was registered with clinicaltrials.gov (NCT01432860). As part of the informed consent process, we explained to potential participants that they were not forced to respond to the questions or wear the device, and they could remove them at any time they felt uncomfortable. To ensure privacy protection, data stored on the Shade sensor were encrypted, and uploaded to an encrypted back-end database. The responses to the completed MUSE surveys were uploaded to a secure back-end database maintained by REDCap [17]. Data were only reviewed by the research team. All data provided to the third party was de-identified.

## Methodology

The self-report and UV sensor measure used in our study, once properly aligned, WILL allow researchers to provide timely interventions to increase awareness of sun exposure to improve sun protection habits. We focus our paper on under- and over-reporting habits which can help us improve our understanding of useful times for interventions. Our framework for aligning self-report and objective sun exposure time comprises four phases. The data collection phase describes the self-report and UV sensor measure. The data pre-processing phase then prepares the data for analysis, filtering self-report and UV sensor readings outside the predefined time. The clustering phase first removes isolated UV sensor events, then groups fragments of sensor readings into single events and removes remaining sensor events that are less than 15 minutes (since participants were not required to self-report events less than 15 minutes). To account for errors in self-report the fourth phase includes the alignment process, where the self-reports are aligned to UV sensor events. We end by presenting the metrics for evaluating the framework at the minute and event level.

### Data collection for self-report and sensor measures

#### Self-report measure

The Daily MUSE Inventory is a computerized measure, administered using REDCap [17], and assesses sun exposure based on the outdoor activities that a participant completed from 6am to 6pm. Each day, participants were asked to report details of all outdoor activities performed for greater than or equal to 15 minutes. Participants first entered an activity description, then they added start and end times, and reported the clothing they were wearing by selecting pictures of clothing options with varying coverage, represented by five pictures each, for four separate body regions (head, torso, legs, and feet). Additional items assessed use of shade provided by trees or shade structures, whether they sweat or got wet, and whether they wore or used each of several accessories (e.g., gloves, hats). Participants then reported all instances of sunscreen use, including the time sunscreen was applied (or reapplied), body sites to which sunscreen was applied, and the SPF of the sunscreen [16].

For the purposes of this paper, the focus is mainly on investigating the accuracy of self-reports of outdoor activity time compared to the sensor data. Hence, only the start and end times of the self-reports along with the activity type are extracted from the MUSE Inventory as described in S1 Fig (refer [16] for complete MUSE Inventory).

#### UV sensor measure of sun exposure time (ground truth)

Most wearable UV sensors use photo-diodes for sensing UVA and UVB to output an electric signal when exposed to UV. Of the many wearable UV sensors, the Shade device [15] was shown to be one of the most accurate and sensitive devices to measure minutes and UV dose (in *joules*/*m*^2^) during outdoor exposure [18]. (Fig 2) The battery lasts five days on a single charge. The sensor is paired to a mobile app (iOS or Android) with data transfer using Bluetooth Low Energy. The non-obtrusive sensor affixes to clothing with a magnetic ring, which makes it easy to wear and prevents damage to clothing. This device maintains an internal data log of accumulated UV dose every six minutes; estimates of exposure minutes are rounded up to the closest multiple of six.

Participants received instruction on how to use the UV sensor and the study smartphone. They were requested to download the Shade sensor data onto the study smartphone every night when they took off the sensor before going to bed. Download time is instantaneous when the phone is in range of a recognized WiFi connection.

### Pre-processing

Across the 39 participants, 2 participants were removed because they reported no outdoor time, despite wearing the UV sensor (thus, there was no data to align). The remaining 37 participants had 290 days of data. A total of 80 days were removed (71 days were removed due to participant noncompliance, minor technical issues, such as dead battery, or lack of sensor wear, 9 days were “true zeros” with no sun activity recorded). All self-reports and sensor events that end before 6am and start after 6pm were removed from analysis, because the ultraviolet index (UVI) is less than 3 [19] in the Midwest before 6am and after 6pm, which is insufficient to cause sunburn. Self-reports shorter than 15 minutes in length were removed since they were inconsistent with the instructions provided (to report events at least 15 minutes in length). Table 1 provides descriptive statistics after pre-processing on the duration of sun exposure events (in hrs.) recorded by participants. Total self-reported time outdoors recorded by the UV sensor exceeded self-reports by 214.22 hours.

**Table 1:** Duration of sun exposure events (in hrs.) recorded by participants (N = 37) using self-report and UV sensor for the days with sun exposure.

	Total (hrs)	Per participant (N = 37)		Per day (N = 290)	
		Mean ± SD	Median	Mean ± SD	Median
**Self report**	439.68	11.88 ± 8.38	9.08	2.05 ± 1.83	1.46
**UV sensor**	653.9	17.67 ± 10.96	16.8	2.27 ± 1.81	1.75

### Sensor data clustering

**Fig 3:**
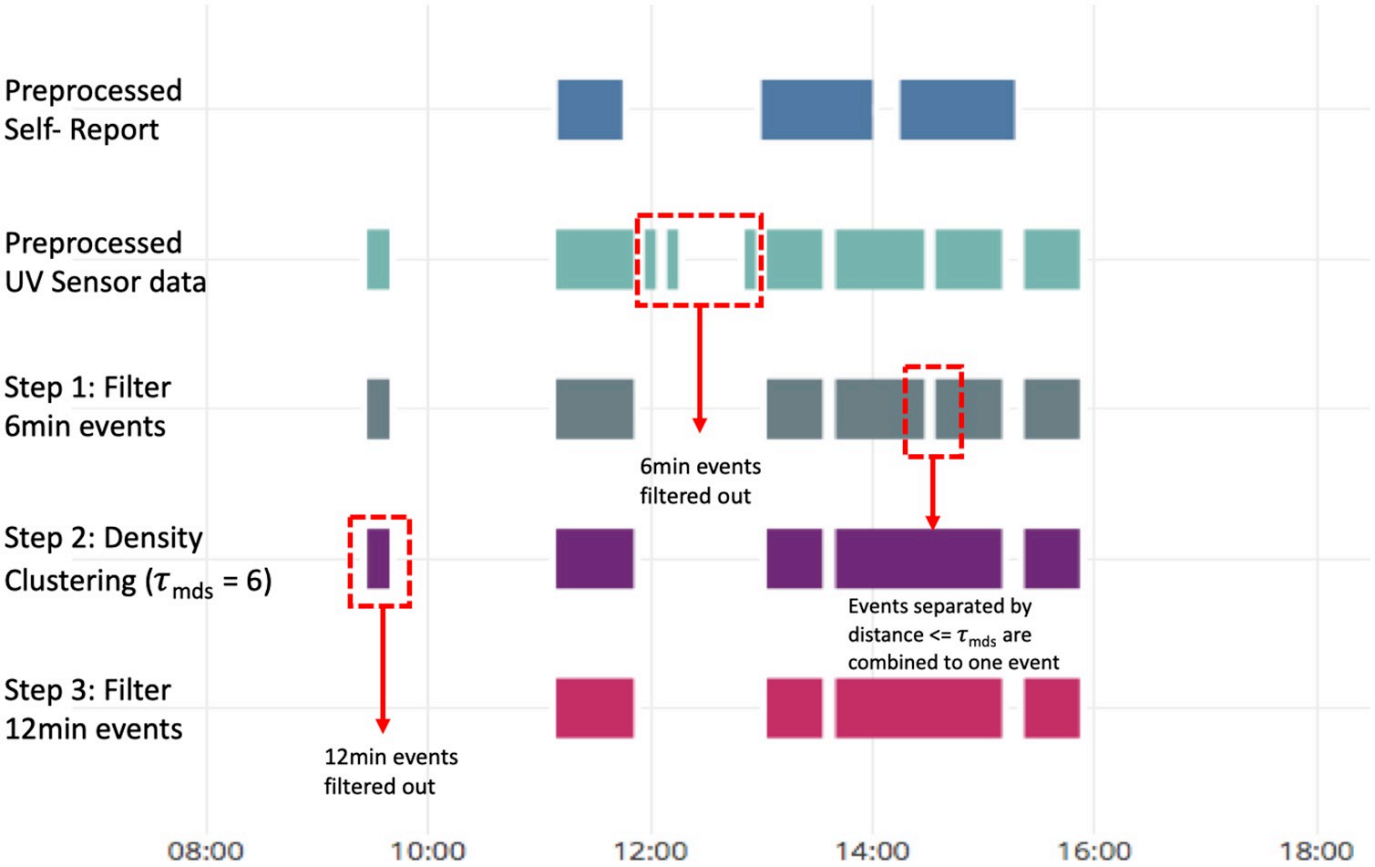
Illustration of one example day for a participant demonstrating the UV sensor data clustering.

To maintain battery lifetime, sensors often collect data at an offset (e.g. every 1 minute, every 6 minutes, every 10 minutes). However, this often results in sensors generating fragments of sensor measurements. As a result clustering of UV sensor measurements is necessary to identify single events. The Shade UV sensor used maintains an internal data log of accumulated UV dose (*J*/*m*^2^) every 6 minutes; estimates of exposure minutes are rounded up to the closest multiple of 6. Due to the fragmentation of sensor measurements and sensitivity of the sensor to UV exposure, clustering of UV sensor measurements is necessary. This process is illustrated in Fig 3. In step 1, the isolated 6-minute sensor events are removed, then, in step 2, the fragments that have a maximum distance of separation (τ_mds_) of 6 minutes are combined together since they may be indicative of a substantial outdoor sun exposure event. Once clustering is applied, any remaining sensor events shorter than the 15-minute minimum duration for self-report are removed (step 3).

### Network flow alignment solution (NFA)

End-of-day self-reports are prone to misalignment in time, due to forgetfulness. For example, a participant reports that they were out in the sun between 9:00am and 9:30am but the sensor records the event between 9:20am and 9:50am. These events are not perfectly aligned and have an offset of 20 minutes. However, from a behavioral standpoint, what matters is that the participant was self-aware that they were out in the sun for 30 minutes somewhere between 9 and 10. In order to give the participant the benefit of the doubt, self-reports are re-aligned with the clustered sensor data by finding an optimal assignment with the objective of minimizing false negative minutes (number of minutes the UV sensor reported a sun exposure for which there is no corresponding self-report).

**Fig 4:**
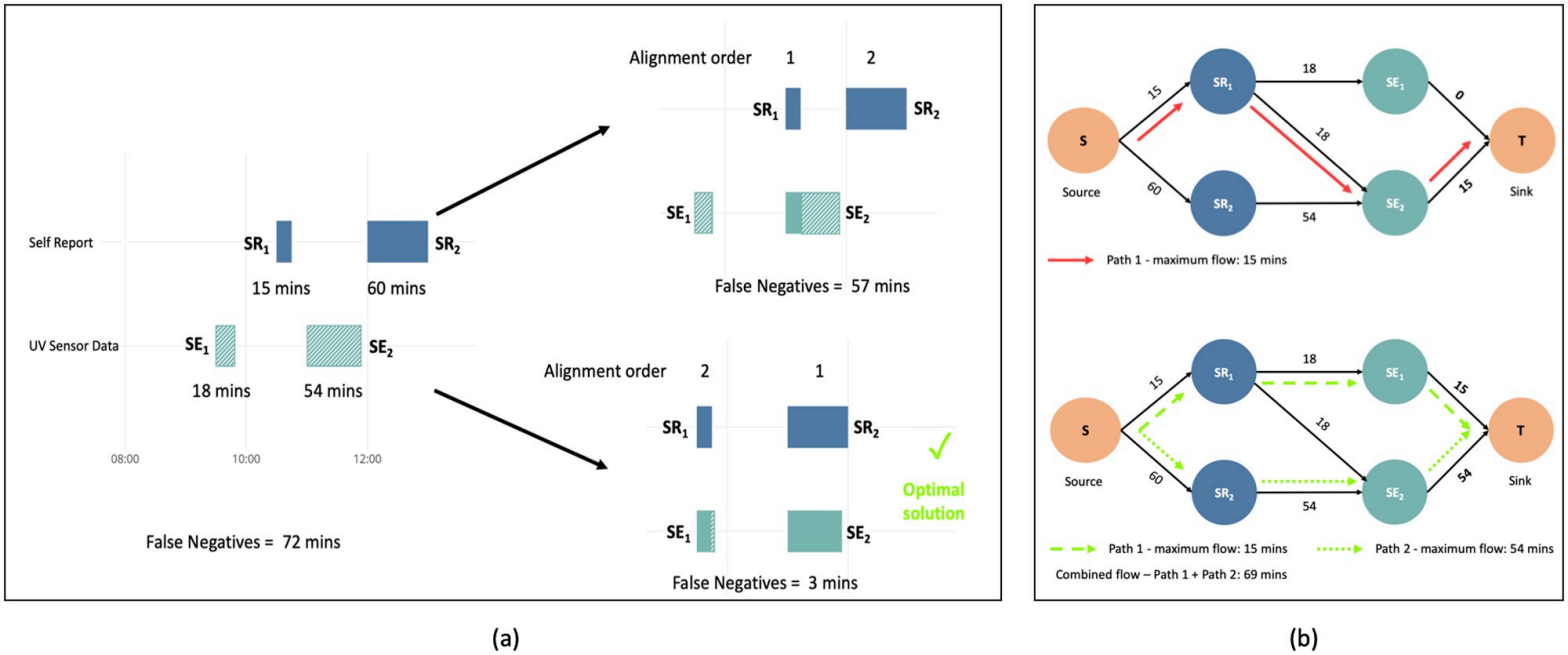
The importance of order in assignment and NFA solution. (a) The left figure shows misalignment of two self-reported events *SR*_1_ and *SR*_2_ with the sensor events. The top right part of the figure shows the reduction in false negative minutes when assigning *SR*_1_ first to its nearest neighbor. The bottom right part of the figure shows what happens when assigning *SR*_2_ first to its nearest unassigned neighbor. (b) Illustration of network flow solution. The top figure shows one path (in red) that enables a flow or alignment of 15 minutes (allowing only 15 units to flow through the first edge from the source to the sink node). Maximum flow is illustrated by the green arrows in the bottom figure, which allows 15 minutes along path 1 and 54 minutes along path 2.

The order of alignment affects the reduction in false negative minutes. In Fig 4a, the first scenario of the 15-minute self-report (*SR*_1_) is aligned to the nearest sensor event (*SE*_2_), followed by the 60-minute self-report (*SR*_2_), there is a false negative reduction of just 15 minutes (from 72 minutes to 57 minutes). In the second scenario, *SR*_2_ is aligned first to the nearest sensor event (*SE*_2_), and then *SR*_1_ is aligned to the next available unassigned sensor event (*SE*_1_). This results in a 69 minute false negative reduction (from 72 minutes to 3 minutes). However, as the number of events increase, finding the correct alignment order becomes computationally challenging since *m* self-reports can yield *m*! assignment orders.

Since individuals are unlikely to misalign events that occur farther apart, we introduce a bounding box within which a self-report can be assigned to a sensor event. The size of the bounding box is set to 60 minutes (the farthest distance a self-report can be assigned to a sensor event). Given a large number of self-reports the exhaustive approach of identifying every possible assignment combination will take more time to compute than necessary, and does not account for a self-report being assigned to more than one sensor event. To solve this problem, we reduce the problem to that of a max-flow min-cut problem. In optimization theory, maximum flow problems are represented using nodes and edges in a graph, where each node is a measurement, and each edge defines a capacity which is the maximum flow the edge can allow to travel from one node to the next. The goal is to find a feasible flow from a source to a sink node in the network such that the flow is maximized [20].

Every self-report and sensor event is represented with a node *SR*_i_ and *SE*_j_, respectively, in the network. A directed edge is defined between every self-report and sensor event it can be assigned to, the capacity of the edges is set to be the duration of the respective sensor events. Fig 4b illustrates how the self-reports and sensor events are represented as a flow network. The goal is to find a path from the source node to the sink node with a maximum flow, representing the maximum number of assigned minutes between self-report and sensor events possible.

### Evaluation metrics

#### Minute-level evaluation metrics

In a 12-hour window (between 6am and 6pm), the minutes where sun exposure was recorded either by the participant in the self-report or by the UV sensor are considered as ‘Positive sun exposure minutes’. The minutes where no sun exposure was recorded by the UV sensor are considered as ‘Negative sun exposure minutes’. By assuming that the UV sensor readings are likely to be ground truth most of the time, the following metrics are defined (depicted in S2a Fig):

1. True positive minutes: Number of minutes in a day where the participant has reported positive sun exposure in the self-report in agreement with the UV sensor data.
2. False positive minutes: Number of minutes in a day where the participant has reported positive sun exposure in the self-report and was recorded as negative sun exposure by the UV sensor. The metric represents over-reporting of sun exposure.
3. False negative minutes: Number of minutes in a day where the participant has reported negative sun exposure in the self-report and was recorded as positive sun exposure by the UV sensor. The metric represents under-reporting of sun exposure.
4. Jaccard: Fraction of true positive minutes over the sum total of true positive, false positive and false negative minutes calculated between the self-report and sensor data. This metric is used to evaluate algorithm performance, see Eq 1.
  $$\begin{array}{c} Jaccard=\frac{TP}{TP+FN+FP} \end{array}$$ (1)

#### Event-level evaluation metrics

In a 12-hour window (between 6am and 6pm), the events where sun exposure was recorded by the UV sensor are considered as ‘Positive sun exposure events’. The events where no sun exposure was recorded by the sensor are considered as ‘Negative sun exposure events’. By assuming the UV sensor events as ground truth, the following are defined (depicted in S1b Fig):

1. True positive self-report: A positive sun exposure event recorded by the participant in the self-report to which there is one or more corresponding events recorded by the UV sensor.
2. False positive self-report: A positive sun exposure event recorded by the participant in the self-report to which there is no corresponding event recorded by the UV sensor. The events that occur between 10am and 4pm are also analyzed, which are considered peak sun exposure times with highest ambient UVI. Further analysis is performed for the false positive self-reports during peak time that yield greater than 30 minutes of sun exposure, which studies have shown result in the greatest impact on sunburn [21].
3. False negative self-report: A positive sun exposure event recorded by the UV sensor to which there is no corresponding self-report completed by the participant. Analysis is performed for all events between 10am to 4pm, and events between 10am to 4pm that are strictly greater than 30 minutes. This shows the times where people may be most inaccurate in self-reporting their outdoor events.

#### Over- and under-reporting in self-reports

After aligning the sensor and self-report data, the number of participants that over- and under-report can be accurately determined. Based on the difference between the total minutes of self-reported outdoor time in a day and the total duration of sensor determined exposure events recorded in a day, each day is classified as an over-reported or an under-reported day. If the difference is greater than 30 minutes (i.e., total minutes of self-report is greater than total minutes of sensor data by at least 30 minutes), the day is classified as an over-reporting day. Otherwise if the difference is less than 30 minutes (i.e., total minutes of self-report is less than total minutes of sensor data by at least 30 minutes), the day is classified as an under-reporting day.

#### Statistical analysis

A paired two-sided t-test was performed on minute-level metrics (Jaccard, true positive, false positive and false negative minutes) to assess whether a significant change was observed after applying the framework (including the clustering and alignment steps).

## Results

### Sensor data clustering

Summary statistics on the duration of events, number of events and UV dosage and number of days at each step of the sensor data clustering process are presented in Table 2. Fig 3 provides a visual representation of the sensor data clustering process for a specific example.

**Table 2:** Overall sun exposure statistics at different stages of sensor data clustering, for a specific example see Fig 3.

	Duration of events (in hrs)	No. of events	No. of days	UV dose (*J*/*m*^2^)
**Pre-processed UV sensor data**	653.9	1712	290	13974.16
**Step 1: Filter 6min events**	594.1	1114	282	13602.59
**Step 2: Density clustering (τ_mds_ = 6)**	605.7	998	282	13602.59
**Step 3: Filter 12min events**	539.5	667	269	12921.85
**Percent change**	17.49%	61.04%	7.24%	7.53%

As shown in Table 2, after completing the sensor data clustering steps, outdoor exposure time was reduced by 17.5%, while UV dose was only reduced by 7.53%. This small reduction in UV dose suggests that our framework does not filter-out biologically-relevant data.

### Minute-level evaluation metrics (algorithm evaluation)

Table 3 summarizes the values for the different minute-level metrics obtained after clustering and aligning the sensor data and self-reports. Following these steps increases the number of true positive minutes, while reducing both false positive and false negative minutes. A statistically significant improvement (*p* < .001) is observed in the Jaccard coefficient and false negative minutes, showing a significant improvement in agreement between self-report and sensor data. While there was a reduction in the false positive minutes and an increase in the true positive minutes after applying the framework, the change was not significant.

**Table 3:** Average minute-level metrics observed for participants (N = 37) at different stages in the UV sensor data clustering and alignment steps. The entire framework ran in 4.1 seconds.

	True positive minutes	False positive minutes	False negative minutes	Jaccard
	Mean ± SD	Mean ± SD	Mean ± SD	Mean ± SD
**Pre-processed UV sensor data**	51.76 ± 34.71	40.13 ± 44.09	79.89 ± 44.72	0.22 ± 0.11
**Step 1: Filter 6min events**	50.87 ± 35.31	42.92 ± 47.12	70.90 ± 41.94	0.24 ± 0.12
**Step 2: Density Clustering (τ_mds_ = 6)**	51.78 ± 35.65	42.01 ± 46.62	72.35 ± 42.95	0.24 ± 0.12
**Step3: Filter 12min events**	51.76 ± 34.53	46.95 ± 51.23	62.38 ± 41.17	0.26 ± 0.13
**Step 4: Alignment (NFA)**	61.76 ± 37.20	36.30 ± 50.12	52.38 ± 37.67	0.36 ± 0.13
**Percent change**	+19.32%	-9.54%	-34.43% *	+63.64% *

* indicates *p* < .001

### Event-level evaluation metrics (over- and under-reporting)

#### Self-report of events

**Fig 5:**
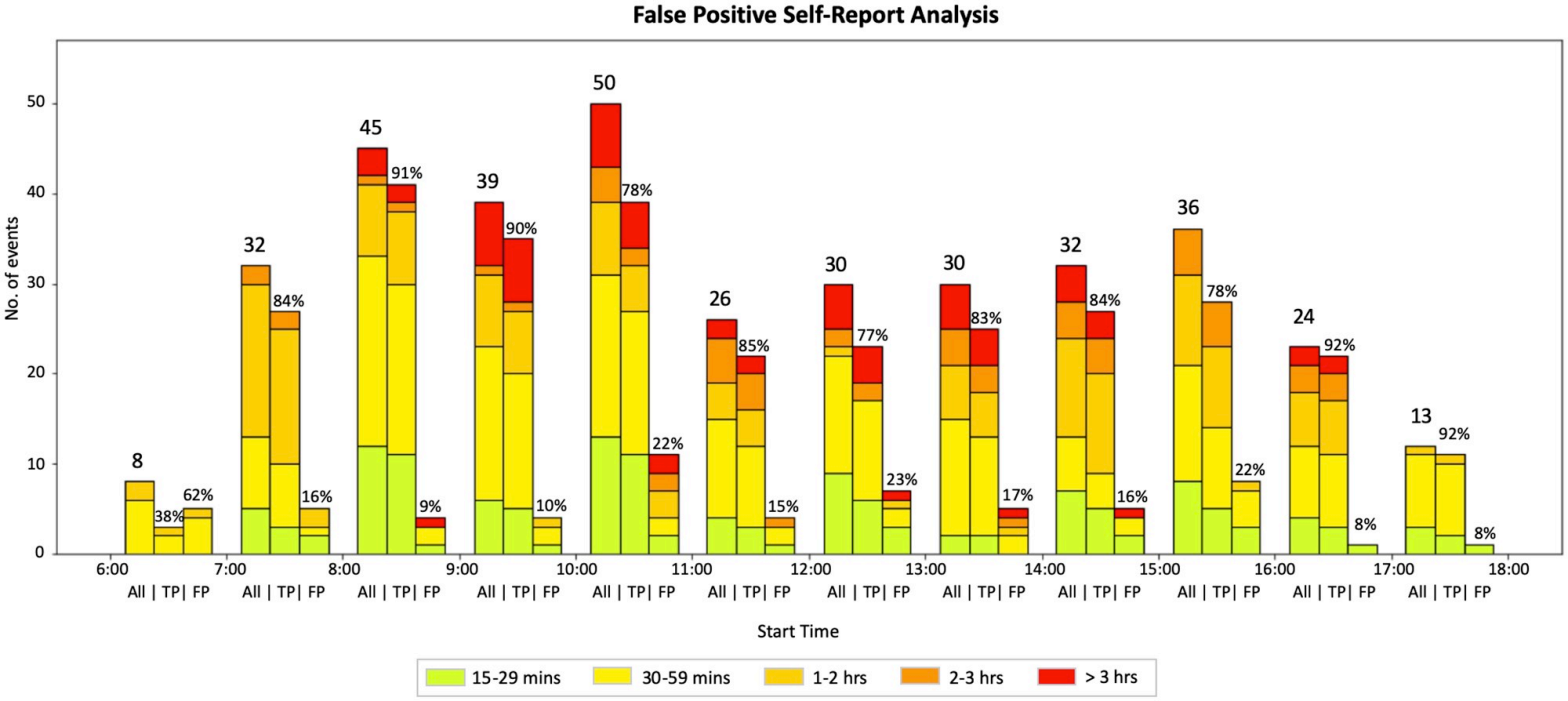
Distribution of all self-reports at the hour level. True positive (TP) and false positive (FP) self-reports (based on the start time of the self-report) are further divided into 5 groups based on their duration: 15-29 minute events, 30-59 minute events, 1-2 hour events, 2-3 hours events, and events longer than 3 hours.

Duration of events were grouped into five intervals. The highest frequency of self-reported activities started between 10am and 11am, with the majority of self-reported activities events that are greater than 3 hours in duration starting between 9am and 11am (Fig 5). In a supplementary analysis, beyond the scope of the present report, we evaluated how activity type impacted false positive self-reports [16].

Table 4 provides statistics on the duration (in mins) of false positive self-reports, which corresponds to over-reporting of time outdoors. The highest rates of over-reporting occurred during peak hours and 45% of the false positive events (28/62) were greater than 30 minutes in duration.

**Table 4:** Statistics on the duration (in mins) of false positive self-reports.

	No. of events	Mean ± SD	Median
**6am–6pm**	62	57.26 ± 60.11	30
**10am–4pm**	41	62.58 ± 59.41	30
**10am–4pm (at least 30 mins of exposure)**	28	84.26 ± 61.43	60

#### UV sensor events

**Fig 6:**
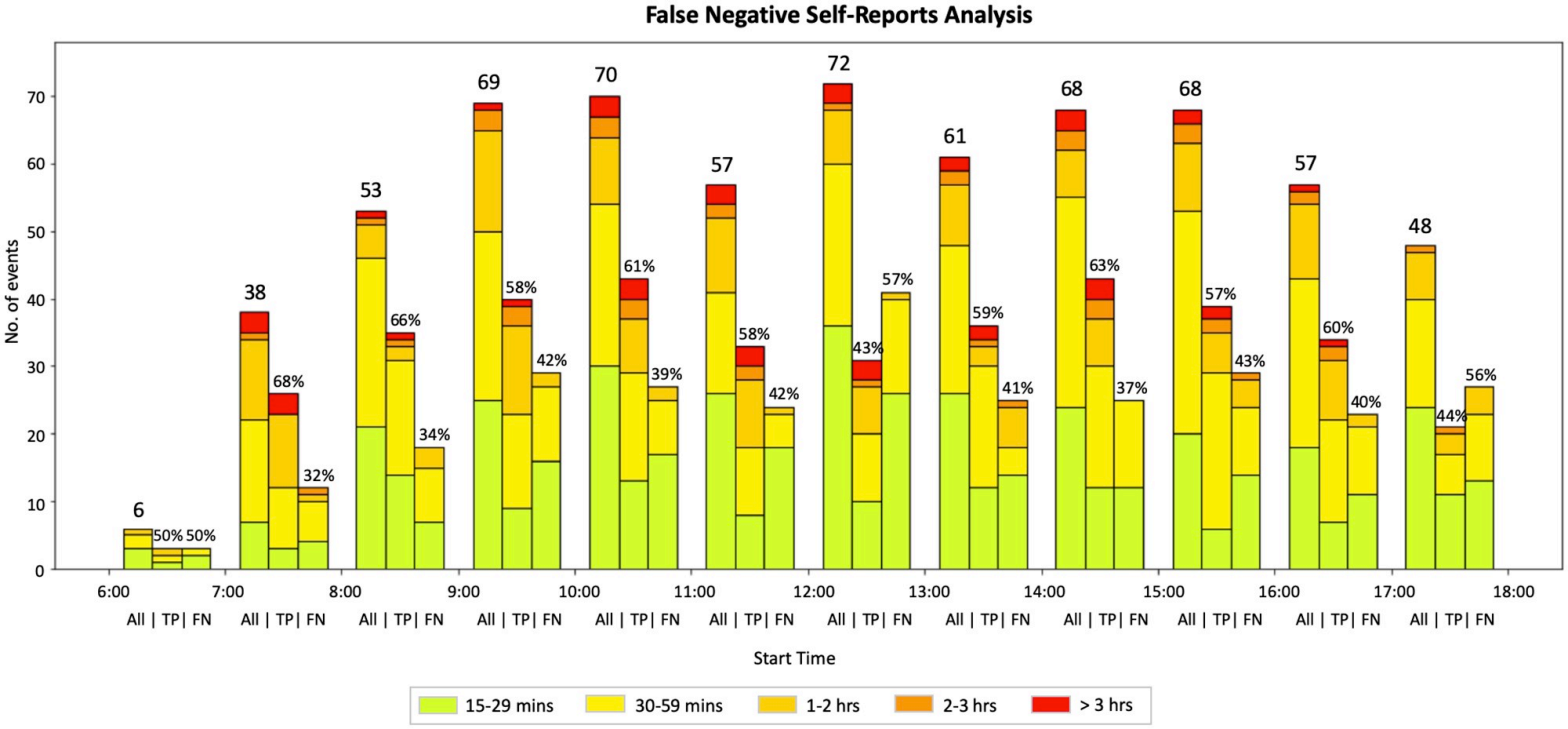
Distribution of all UV sensor events at the hour level. Sensor events corresponding to true positive (TP) and false negative (FN) self-reports (based on the start time of the sensor event) are further divided into 5 groups based on their duration: 15-29 minute events, 30-59 minute events, 1-2 hour events, 2-3 hours events, and events longer than 3 hours.

The highest frequency of events started between 9-11am, 12-1pm and 2-4pm. (Fig 6) The largest portion of events 2-3 hours in duration are reported to start at 9-11am.

Table 5 provides statistics on the duration (in mins) of sensor events corresponding to the false negative self-reports. 62% (172 out of 279 events) of the sensor events corresponding to the false negative self-reports started between 10am and 4pm, and 40% (68 out of 172 events) of the peak hour false negative self-reports are greater than 30 minutes in duration.

**Table 5:** Statistics on the duration (in mins) of sensor events corresponding to false negative self-reports.

	No. of events	Mean ± SD	Median
**6am–6pm**	279	32.06 ± 20.11	24
**10am–4pm**	172	27.55 ± 11.58	24
**10am–4pm (at least 30 mins of exposure)**	68	43.02 ± 16.22	36

Since the largest percentage of false negative self-reports occurs between 12 and 1pm (57% of the sensor events within this hour have no corresponding self-report), the risk of sunburn is amplified by the intensity of the sun at this time. The second highest percentage of false negative self-reports is between 5 and 6pm (56% of sensor events within this hour have no corresponding self-report).

### Over- and under-reporting in self-reports

**Fig 7:**
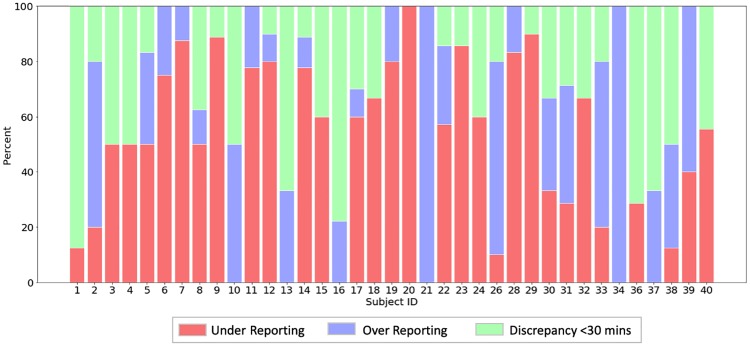
Over- and under-reporting in self-reports at the participant-level (N = 37). Each bar shows the percentage of days (after pre-processing and removing days with missing data) where the participant over- or under-reported, or the self-reported minutes were equivalent to the sensor reports (i.e. discrepancy was within 30 minutes).

Table 6 provides statistics on the number of days and minutes where the participants over- and under-reported their time outdoors in self-reports after applying the clustering and NFA algorithm. Fig 7 shows, for each participant, the fraction of days where they over-reported, under-reported, or the sensor and self-reported minutes were equivalent (i.e. discrepancy was within 30 minutes). Only one participant exclusively under-reported and two participants exclusively over-reported during the course of the study. A total of 24 participants over-reported on at least 1 day, and 31 participants under-reported on at least 1 day. There were 18 participants who both under- and over-reported over the course of the study.

**Table 6:** Days and the number of minutes where participants over- and under-reported their sun exposure in their self-reports.

	No. of days (% days)	Total discrepancy (in mins)	Avg. discrepancy per day (in mins)
			Mean ± SD
**Over-reporting (at least 30 false positive minutes per day)**	58 (21%)	7,136	123.03 ± 105.62
**Under-reporting (at least 30 false negative minutes per day)**	138 (51%)	13,123	95.09 ± 64.84

### Exit interviews

An exit interview identified that adult melanoma survivors were willing to wear the UV sensor despite not receiving daily feedback during this observational study. (Table 7) The high adherence of melanoma survivors to wearing the sensors may be related to their anticipation of receiving a final report of their UV exposure and incentives.

**Table 7:** Summary of the responses provided by participants during the exit interviews.

Variable	Participants (n = 39)	Circumstance / characteristics (n)
Willing to wear/use when not part of research ^a^ (acceptable features)	Yes (34)	Could forget they were wearing it (8) Easy to download/wear (17) Vibration when covered a good reminder (5)
	No (5)	Only wear it when habits change
(Barriers to wear) Difficulty wearing sensor ^a^	Reported (36)	Too big/ bulky/heavy (17) Gets in the way of purse, seat-belt (9) Vibration annoying (6) Appearance (unattractive, looks like camera) (6) Clothing pulled by weight/ can’t wear jacket (4)

^*a*^ Participants specifically probed for item. Participants provided more than one response.

## Discussion

To better understand why melanoma survivors often engage in risky sun exposure, in this study, we examined survivors’ behavioral awareness for time spent outdoors. We built and applied an algorithm that allowed for alignment of self-report and UV sensor events, after accounting for variation in each measure (e.g., minor offsets in time) that we do not view as meaningful for assessing behavioral awareness. Our use of UV sensors and self-reports of outdoor time over a 10-day period allowed not only identification of periods of potentially risky sun exposure but also assessment of the prevalence and time frames during which a sample of melanoma survivors tended to under-report their sun exposure. After applying clustering and alignment algorithms to the UV sensor and self-report data, the outdoor times reported in both measures were compared. Under-reporting of sun exposure was operationalized as “false negative minutes”—sensor-assessed minutes/events of exposure which have no corresponding self-report. Under-reporting of sun exposure time occurred on 51% of the days analyzed and more participants tended to under-report than to over-report sun exposure time. Rates of under-reporting of sun exposure were highest for events that began from 12-1pm, and second-highest from 5-6pm. Using sensors to provide participants feedback regarding their exposure during peak hours of UV intensity may help reduce sun exposure, especially for instances when they do not accurately recall their outdoor time.

The 12-1pm time period during which the highest rates of under-reporting of sun exposure were observed is particularly important since it occurs during the peak hours when UV rays are strongest and sunburns are most likely. The time frame and average duration of these under-reported events suggests that individuals could be getting more sun exposure during mid-day breaks and late afternoon-evening periods when they may be commuting than they may recall. However, these times may also be times of intentional exercise or physical activity, which is an important target of research, particularly among melanoma survivors. Individuals are likely not completely unaware of their outdoor time during these windows—more likely, underestimating how long they were outside and choosing not to report the activity in the end-of-day surveys, which specify that participants should only record activities that were 15 minutes or longer. These time-points may represent important targets for sun exposure intervention.

Our adherence data and exit interview findings indicate that a sensor-based study for sun exposure is a feasible option. Despite the identified barriers to wearing the sensor (e.g., getting in the way of seat-belts and straps such as on backpacks), most participants who wore the sensor reported wearing the sensor during waking hours for 10 days of the study. Although this high degree of compliance was likely driven by the research instructions, incentives, and the participants being melanoma survivors, participants indicated in their exit interviews they would be willing to use a sensor outside of a research study and receive alerts and advice based on the data it recorded. Most barriers to wearing the sensor were related to its appearance and size, which may be addressed in future studies in order to enhance adherence with wearing the sensor.

The analysis of under-reporting of sun exposure time was made possible through the use of a robust network flow alignment (NFA) algorithm that temporally aligns exposure event reported in self-report and UV sensor. The run time of the algorithm—4.1 sec for this data set—will allow real-time use for timely user feedback to improve sun protected outdoor activities, and prevent sunburn.

This study builds upon previous research comparing self-reports of sun behavior to objective measures [22–24] by 1) evaluating the specific types of error that participants make when self-reporting behavior and 2) analyzing discrepancies at a fine-grained level (individual events, day). In most cases, comparisons between self-reports of sun behavior and objective measures are performed for the purposes of validating the self-report measure, as we had also done for the MUSE Inventory. Such validation studies typically find good (but not excellent) agreement, indicating that the measures are adequate, but measurement error remains that could provide insights into participants’ awareness of their own sun exposure. In this study, we sought to better understand not just the level of agreement between measures but also the nature of differences between self-report and the objective measures. Understanding whether participants are more likely to over- or under- report exposure, the times of day discrepancies are most likely, and the specific activities that contribute to discrepancies may provide valuable information for interventions.

### Limitations

This study had several limitations that we will address in future research. First, the existing online MUSE self-report measure requires participants to report at the end-of-day. Future studies will look at the feasibility of integrating the self-report measure into a smartphone app to improve timely reporting of sun protection.

Second, although the UV sensor is treated as “ground truth” in this study, it is an imperfect measure of outdoor time. Applying the framework, improved agreement, yet overall agreement remains low (i.e. Jaccard of 0.36 on average across all participants) between self-report and sensor data. By applying the framework, we are able to address the challenges in the data collected and facilitate a comparison of the sensor and self-report measures. Removing some of the “noise” between the measures provides greater confidence that differences between measures indicate a meaningful lack of awareness of sun exposure rather than reflecting measurement challenges such as those depicted in Fig 1. While not as common as under-reporting, over-reporting, in which an individual self-reported an event for which there was no corresponding sensor event, also occurred on 21% of the days. A possible explanation of over-reporting may be due to the limitations of a UV sensor, which can become shaded by the participants’ own body or ambient shade, and it, thus, may not capture all their outdoor exposure time.

Third, the participants enrolled in the study were limited to a small number of melanoma survivors from the Midwest United States during the summer days.

### Future work

In order to obtain a more definitive measure of outdoor time, wearable cameras may serve as promising future direction. Of note, the removal of the sensor data by clustering did not result in loss of meaningful total UV exposure as shown in Table 2, which shows the robustness of our technique. While we justify this approach on the basis of a review of descriptive data patterns in the present sample, to truly determine the optimal value, an absolute ground truth metric is needed, such as footage from a wearable video camera. Unfortunately, wearable video cameras are subject to privacy concerns [25], and as a result, techniques such as ours are needed to merge between objective and subjective measures. However, future work with privacy preserving wearable cameras will further improve our ability to more objectively align between sensor and self-report measures.

Future work will also explore other combinatorial optimization techniques such as the generalized assignment problem [26] to further optimize alignment. Our current framework may assign a self-report to two different UV sensor events, because some flow may be sent to one sensor event, while the rest travels to another sensor event. While counter intuitive, this result may be consistent with actual behavior, given the potential for participants to be shaded outdoors for some time, potential forgetfulness, and the nature of the UV sensor event detection. We will also explore other types of costs and profits to displacing self-reports, where the cost may be weighted by the time of day, and the profit could be based on the type of activity.

Another area of future work is the duration of the bounding box, which determines the allowed assignment between self-reports and UV sensor events. This framework can also be used at longer duration’s of participant self-report recall, at the week or month level. Adjusting the bounding box can allow for assignments to occur at farther distances from the self-report, based on the participants’ expected recall potential.

Lastly, responses to the exit interview questions suggested ways in which alerts based on the UV sensor might be most effectively implemented in future interventions. Since participants found it difficult to estimate the amount of UV exposure on a cloudy day, it would be worth having the sensor alert them about their UV exposure when engaged in outdoor activities on a cloudy day. Another potential alert would be during outdoor activities performed during the period of peak UV intensity.

## Conclusion

Despite their high recurrence risk, we found that melanoma survivors are often exposed to the sun during peak times and often under-report their sun exposure time. This analysis of behavioral recall was made possible by using a novel framework for processing UV sensor data and aligning self-reports to better assess self-report recall. By applying our framework, we observe a significant reduction in the false negative minutes (34.43%) and a significant improvement in Jaccard by 63.64%. This effort sheds light on the potential for wearable passive sensors and self-report data to be used together to understand participant behavior and continuously optimize behavioral interventions to improve sun protection among at-risk patients.

## Supporting information

S1 Fig Muse Inventory. (TIF)

S2 Fig Illustration of the different evaluation metrics. (a) Minute-level (b) Event-level.
(TIF)
