# Supplementary figures and images for: Assessing recall of personal sun exposure by integrating UV dosimeter and self-reported data with a network flow framework
Source: PLoS One. 2019 Dec 4;14(12):e0225371. doi: 10.1371/journal.pone.0225371 (PMC6892536; doi:10.1371/journal.pone.0225371)

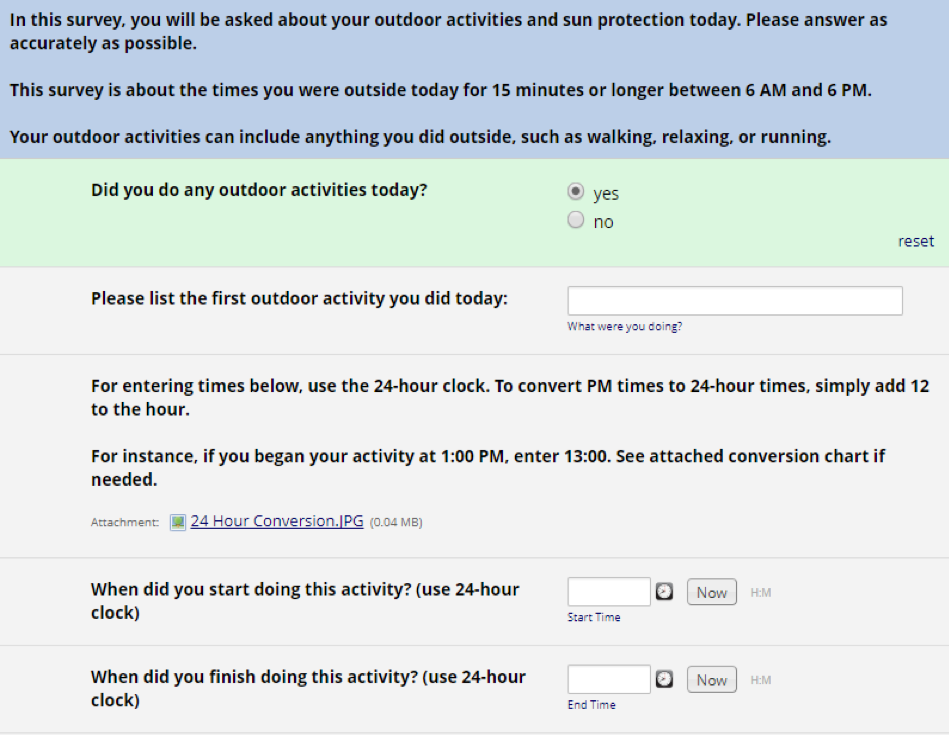

Supplement: S1 Fig — (TIF) [file pone.0225371.s001.tif]

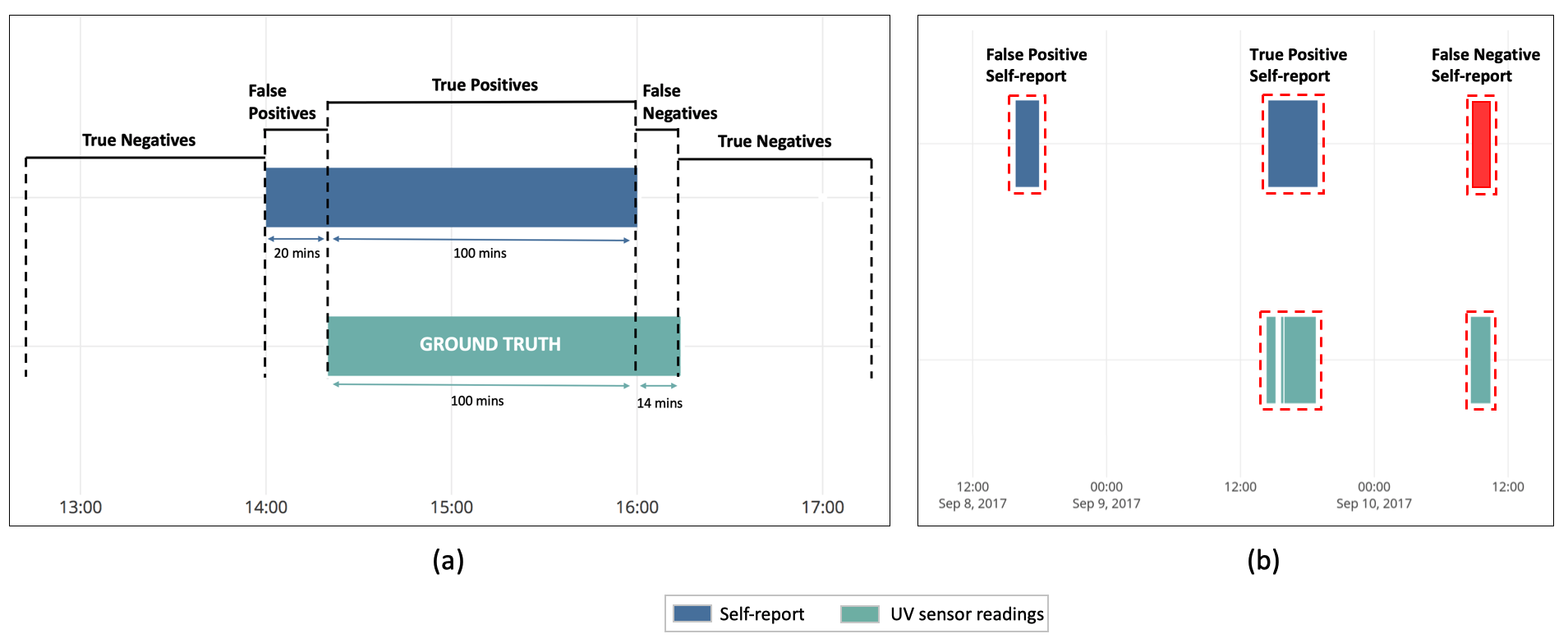

Supplement: S2 Fig — (a) Minute-level (b) Event-level. (TIF) [file pone.0225371.s002.tif]
